# Editorial for the Special Issue on Plant Polyphenols in the Immune and Inflammatory Responses

**DOI:** 10.3390/biom13050814

**Published:** 2023-05-11

**Authors:** Heping Cao

**Affiliations:** United States Department of Agriculture, Agricultural Research Service, Southern Regional Research Center, New Orleans, LA 70124, USA; heping.cao@usda.gov; Tel.: +1-504-286-4351

Inflammation and associated immune diseases have placed a heavy burden on health care systems [1,2]. Drug treatment for reducing inflammation and related diseases has not been satisfactory. Therefore, complementary and alternative approaches need to be evaluated [3].

Plant extracts have historically been used as alternative medicines for the prevention, alleviation, and cure of various diseases [4,5]. The mechanisms of how bioactive plant extracts work are poorly understood due in part to the lack of knowledge on the structures of bioactive components in most of these extracts. 

Plant polyphenols are major bioactive compounds in plant extracts [6,7]. They are produced through the flavonoid biosynthetic pathway in plants and used naturally by plants for defenses against predators. Plant polyphenols are present in most diets and beneficial to human health [8,9,10]. They have been found to regulate mammalian gene expression in numerous studies [11,12,13,14,15,16,17,18,19,20,21]. Anti-inflammatory activities of plant polyphenols are proposed to play an important role in the mediation of various health conditions via these alternative therapies; however, their anti-inflammatory mechanisms are not completely understood [22,23,24].

This Special Issue aims to highlight plant polyphenols in the immune and inflammatory responses. The topics include plant polyphenol extraction, identification and bioactivity; structure–function relationship; and molecular mechanisms at the DNA, RNA, protein and metabolic levels. This Special Issue consists of five papers including three comprehensive reviews and two original research articles.

(1) “Therapeutic Potential of Leaves from *Fridericia chica* (Bonpl.) L. G. Lohmann: Botanical Aspects, Phytochemical and Biological, Anti-Inflammatory, Antioxidant and Healing Action” [25]. In this paper, Dr. Sartim et al. examined the medicinal plant of the species *Fridericia chica* (Bonpl.) L. G. Lohmann (Bignoniaceae), which is widely distributed in Brazil and known as a traditional folk medicine for the treatment of intestinal colic, diarrhea, and anemia, among other diseases. The leaf extracts were found to contain many types of plant phenolic compounds. The authors summarized the data from several studies on the therapeutic efficacy of *F. chica* extracts, including antitumor, antiviral, wound healing, anti-inflammatory and antioxidant activities. The healing action of *F. chica* leaf extract was demonstrated in several experimental models, with the ability to increase the proliferation of fibroblasts for tissue repair. The anti-inflammatory activity of *F. chica* was found to be related to 3-deoxyanthocyanidins, which are capable of inhibiting pro-inflammatory pathways such as the kappa B (NF-kB) nuclear transcription factor pathway. The antioxidant effect was attributed to phenolic compounds interrupting chain reactions caused by free radicals and donating hydrogen atoms or electrons. The authors concluded that the species *Fridericia chica* has great therapeutic potential, and the information presented could encourage new research and promote plant phenolic extracts as herbal medicines in health care systems.
(2) “Renoprotective Effects of Luteolin: Therapeutic Potential for COVID-19-Associated Acute Kidney Injuries” [26]. Dr. Sousa and coauthors summarized the progress made regarding luteolin’s renoprotective effects on acute kidney injury (AKI) in critically ill COVID-19 patients. To date, there is no specific treatment for COVID-19 and its associated AKI. Luteolin is a natural compound with multiple pharmacological activities, including anticoronavirus and renoprotective activities against kidney injury induced by sepsis, renal ischemia and diverse nephrotoxic agents. The authors mechanistically discussed the anti-SARS-CoV-2 and renoprotective activities of luteolin and highlighted its therapeutic potential for the treatment of AKI in COVID-19 patients.
(3) “The Ethnopharmacological Uses, Metabolite Diversity, and Bioactivity of Rhaponticum uniflorum (Leuzea uniflora): A Comprehensive Review” [27]. Dr. Olennikov analyzed the scientific literature from 1991 to 2022 on *Rhaponticum uniflorum* (L.) DC. (*syn*. *Leuzea uniflora* (L.) Holub), which is widely used as an anti-inflammatory and stimulant remedy in Asian traditional medicines, including in China, Siberia and Mongolia. The chemodiversity of *R. uniflorum* contains 225 compounds, including sesquiterpenes, ecdysteroids, triterpenes, sterols, thiophenes, hydroxycinnamates, flavonoids, lignans, nucleosides and vitamins, alkanes, fatty acids and carbohydrates. Plant phenolics (76 compounds) and triterpenoids (69 compounds) are the most studied groups of phytochemicals. This review summarized the information on the methods of chromatographic analysis of selected compounds and the quantitative content of some components in various organs of *R. uniflorum*. The published results show that the plant extracts and some of the compounds have a wide range of biological activities, including anti-inflammatory, antitumor, immunostimulatory, anxiolytic, stress-protective, actoprotective, antihypoxic, anabolic, hepatoprotective, anti-atherosclerotic and hypolipidemic properties, along with the inhibition of PPARγ receptors. The author pointed out that the potential medicinal application of this plant’s extract and its compounds requires further clinical studies.
(4) “Influence of Diets Enriched with Flavonoids (Cocoa and Hesperidin) on the Systemic Immunity of Intensively Trained and Exhausted Rats” [28]. Dr. Castell and colleagues studied the influence of flavonoid-enriched diets on the immune alterations induced by an intensive training and a final exhaustion test in rats. A flavanol-enriched diet (C10 diet) and a flavanol and flavanone-enriched diet (CH diet) were used. Lewis rats were fed either a standard diet, the C10 diet or the CH diet and submitted to an intensive running training for six weeks on a treadmill. The C10 diet attenuated the increase in plasma cortisol induced by exhaustion, while both the C10 and CH diets prevented the alterations in the spleen Th cell proportion. The experimental diets also induced an increase in serum immunoglobulin concentration and an enhancement of spleen natural killer cytotoxicity, which may be beneficial in cases with a weakened immunity. Most of the effects observed in the CH groups seemed to be due to the cocoa content. Their results indicate that a dietary intervention with flavonoids enhances immune function, partially attenuating the alterations in systemic immunity induced by intensive training or exhausting exercise.
(5) “Plant Polyphenol Gossypol Induced Cell Death and Its Association with Gene Expression in Mouse Macrophages” [29]. Gossypol is a complex plant polyphenol with six OH groups and six CH_3_ groups in its molecule, which is found in the small intercellular pigment glands in cotton plants, especially in glanded cottonseed. Drs. Cao and Sethumadhavan reported the effects of gossypol on cell viability and gene expression in mouse macrophages. They explored gossypol’s toxicity and its effect on gene expression involved in the inflammatory response, glucose transport and insulin signaling pathways in mouse RAW264.7 macrophages after being treated with multiple concentrations of gossypol for 2–24 h. Their results showed that cell viability was substantially reduced by gossypol, which was accompanied with a dramatic reduction in soluble protein content in macrophages. Gossypol treatment significantly increased the mRNA levels of anti-inflammatory TTP genes. However, gossypol also increased proinflammatory cytokine mRNA levels, including TNF, COX2, GM-CSF, INFγ and IL12b. Gossypol treatment upregulated mRNA levels of GLUT1, GLUT3 and GLUT4 genes, as well as INSR, AKT1, PIK3R1 and LEPR, but not APP genes. This study demonstrated that gossypol induced macrophage death and reduced soluble protein content, which was accompanied with the massive stimulation of anti-inflammatory TTP family and proinflammatory cytokine gene expression, as well as the elevation of gene expression involved in glucose transport and the insulin signaling pathway in mouse macrophages.
